# Physiological Responses of *Robinia pseudoacacia* and *Quercus acutissima* Seedlings to Repeated Drought-Rewatering Under Different Planting Methods

**DOI:** 10.3389/fpls.2021.760510

**Published:** 2021-12-06

**Authors:** Xiao Liu, Qinyuan Zhang, Meixia Song, Ning Wang, Peixian Fan, Pan Wu, Kening Cui, Peiming Zheng, Ning Du, Hui Wang, Renqing Wang

**Affiliations:** ^1^Institute of Ecology and Biodiversity, School of Life Sciences, Shandong University, Qingdao, China; ^2^Shandong Provincial Engineering and Technology Research Center for Vegetation Ecology, Shandong University, Qingdao, China; ^3^Qingdao Forest Ecology Research Station of National Forestry and Grassland Administration, Shandong University, Qingdao, China; ^4^Qingdao Forestry Station, Qingdao, China

**Keywords:** compensatory mechanism, drought resistance ability, mixed planting, plant hydraulic traits, stress imprint, warm temperate zone

## Abstract

Changing precipitation patterns have aggravated the existing uneven water distribution, leading to the alternation of drought and rewatering. Based on this variation, we studied species, namely, *Robinia pseudoacacia* and *Quercus acutissima*, with different root forms and water regulation strategy to determine physiological responses to repeated drought-rewatering under different planting methods. Growth, physiological, and hydraulic traits were measured using pure and mixed planting seedlings that were subjected to drought, repeated drought-rewatering (i.e., treatments), and well-irrigated seedlings (i.e., control). Drought had negative effects on plant functional traits, such as significantly decreased xylem water potential (Ψ_md_), net photosynthetic rate (A_P_), and then height and basal diameter growth were slowed down, while plant species could form stress imprint and adopt compensatory mechanism after repeated drought-rewatering. Mixed planting of the two tree species prolonged the desiccation time during drought, slowed down Ψ_md_ and A_P_ decreasing, and after rewatering, plant functional traits could recover faster than pure planting. Our results demonstrate that repeated drought-rewatering could make plant species form stress imprint and adopt compensatory mechanism, while mixed planting could weaken the inhibition of drought and finally improve the overall drought resistance; this mechanism may provide a theoretical basis for afforestation and vegetation restoration in the warm temperate zone under rising uneven spatiotemporal water distribution.

## Introduction

Precipitation patterns have been substantially altered as a consequence of global climate change, specifically, the frequency and intensity of precipitation increase, but the total amount decreases (Easterling et al., 2000; Högy et al., 2013; Gimbel et al., 2015; Ge et al., 2017). This phenomenon has aggravated the existing spatiotemporal uneven water distribution in the warm temperate zone (Luan et al., 2011; Corlett, 2016), leading to the alternation of drought and rewatering. Severe drought may lead to plant hydraulic failure, which has been invoked as the most direct and critical mechanism causing tree dieback and forest mortality (Martinez-Vilalta and Pinol, 2002; Nardini et al., 2013; O’Grady et al., 2013; Liu et al., 2020). After drought, the pace of recovery differs among physiological processes; leaf water potential typically recovers within few days even few hours upon rewatering, while leaf gas exchange-related variables lag (Ruehr et al., 2019), leading to plant growth traits slow recovery (Li et al., 2019, 2020).

Drought and rewatering will be likely repeated (Tomasella et al., 2019). There are two main strategies for plants to respond to repeated drought-rewatering. One is stress imprint, which means that the history of exposure to stresses will change the subsequent responses of plant species, make them more resistant, and respond faster to future stresses (Walter et al., 2013; Forner et al., 2018; Tomasella et al., 2019). Main mechanisms of stress imprint are storage of stress-resistant substances through physiological and biochemical responses, accumulation of signal proteins or transcription factors, and epigenetic changes caused by DNA methylation (Bruce et al., 2007; Liu and He, 2020). The other is pressure fatigue, which refers to the phenomenon that under cyclic stress, plant functional traits cannot be completely recovered, gradually weaken, or even lose (Umebayashi et al., 2019), such as under repeated drought, hydraulic conductivity could not recover to the origin level after rewatering (Moran et al., 2017). In that way, in the warm temperate zone, which is the main strategy for plant species to respond to the repeated drought-rewatering?

Ecological theories suggest that multigroup composite structures of community often show large differences in ecological strategies for coping with environmental stress (Richards et al., 2010; Forrester and Bauhus, 2016). Some previous studies have reported contradictory findings about the relationship between plant diversity and drought. Lebourgeois et al. (2013) and Pretzsch et al. (2013) show that the mixed-species forests are divided by hydrological niches, which could reduce the competition for limited water resources and strengthened regional drought resistance. Such as in mixed planting, the differences in the distribution and structure of plant roots of different species lead to less overall competition for water than that in pure planting (Schwendenmann et al., 2015), which may prolong the desiccation time of drought (Blackman et al., 2019). However, if the interacting species have similar functional characteristics (i.e., functional redundancy), ecological niche overlap may lead to increasing water competition, which in turn increases the degree of drought. Therefore, it is important to understand how mixed-species forests of specific species in a limited space to cope with drought together. However, due to the multiple interrelated processes and complex feedback mechanisms between plant diversity and drought stress, it is still unknown whether mixed-species forests can attenuate regional drought stress.

*Robinia pseudoacacia* L., shallow-rooted species with wide root crown width, and *Quercus acutissima* Carr., deep-rooted species with narrow root crown width, are dominant species in the tree layer of temperate deciduous broadleaved forests in Northern China. They are widely dispersed sympatric tree species in Northern China (Wang and Zhou, 2000; Wang et al., 2020), and in recent research, they are renowned for their high drought tolerance and fast recovery capability in the warm temperate zone (Xu et al., 2009; Du et al., 2017; Li et al., 2019; Wang et al., 2020). Moreover, on the “isohydric-anisohydric” continuous spectrum, *R. pseudoacacia* is an anisohydric species, while *Q. acutissima* is an isohydric one (Wang and Zhou, 2000; Li et al., 2020). Besides, there are many seedlings in the regeneration layer under forests (Wang et al., 2021). The regeneration of woody plant seedlings is an important component of maintaining the vegetation diversity of forest ecosystem (Fukushima et al., 2008), playing a central role in the process of community assembly and forest dynamic changes (Nyland et al., 2006). Therefore, we set up a repeated drought-rewatering experiment to find out how plant functional traits of *R. pseudoacacia* and *Q. acutissima* respond to repeated drought and rewatering under different planting methods. We hypothesized that (1) the first drought may make the two plant species form stress imprint, resulting in fast recovery of plant functional traits after repeated drought-rewatering and (2) mixed planting may weaken the negative effects of drought.

## Materials and Methods

### Plant Materials

This study was conducted at the Fanggan Research Station at Shandong University in Jinan, Shandong Province, China (36°26′ N, 117°27′ E). Seeds from *R. pseudoacacia* L. were collected from the trees in our common garden, while seeds from *Q. acutissima* Carr. were collected from the trees on a mountain nearby, and they were stored at 4°C in a refrigerator. These seeds were germinated in a growth chamber filled with humus soil in early April 2018. When most seedlings reached 10 cm, healthy and uniform germinants were sown in plastic pots (32 × 29 cm, height × diameter) with an 8 kg mixed sandy loam and humus soil, whose holding capacity of soil water at full saturation was ∼2 kg. Seedlings in the pots were allowed to grow for 3 months. The average greenhouse conditions for the entire duration after treatments were as follows: air temperature 30.2°C (26.7–33.5°C) in the daytime (06:00–18:00) and 24.8°C (20.2–28.6°C) at night (18:00–06:00) and relative humidity 79.3% (55.2–100%) in the daytime and 93.6% (67.3–100%) at night.

### Experimental Design

Two planting methods were used in our research, namely, pure planting, two *R*. *pseudoacacia* in one pot (RR) or two *Q. acutissima* in one pot (QQ), and mixed planting, one *R*. *pseudoacacia* (R_Q_) and one *Q. acutissima* (Q_R_) in the same pot. In both planting methods, two seedlings were planted at one-third and two-thirds of the pot diameter, respectively. Each planting method had 72 well-irrigated pots of seedlings before treatments. In control group (CK), 28 pots had been well irrigated to the end of the experiment; in drought treatment (D), when treatments began, first, 44 pots were withheld from watering on July 1 for 10 days; second, 20 out of the 44 drought treatment pots underwent the first rewatering treatment (R_1_) on July 11 for 20 days; and then, 12 out of the 20 that were given first rewatering treatment pots underwent repeated drought treatment on July 31 for 10 days; finally, 8 out of the 12 pots underwent repeated rewatering treatment (R_2_) on August 10 for 20 days. Both R_1_ and R_2_ belonged to repeated drought-rewatering treatment (R). We drew a table to summarize our experimental design (Table 1). In our experiment, 10 days after drought resulted in soil water content of all pots reaching *c.* 20% of the field capacity (2 kg). We had seven harvest times, namely, July 1 (Day 0), July 11 (Day 10), July 21 (Day 20), July 31 (Day 30), August 10 (Day 40), August 20 (Day 50), and August 30 (Day 60). On July 1, we only harvested the control group; on July 11, we harvested both control group and drought treatment; on other harvest times, we harvested all the groups. Four pots of each planting method and treatment were randomly selected for measurement.

**TABLE 1:** Table for experimental design. Data stand for remaining pots at harvest times of each treatment before harvesting.

	Before treatments		First drought-rewatering			Repeated drought-rewatering		
H	Before Jul. 1st	Jul. 1st	Jul. 11th	Jul. 21st	Jul. 31st	Aug. 10th	Aug. 20th	Aug. 30th
CK	72	28	24	20	16	12	8	4
D	–	44	24	20	16	12	8	4
R	–	–	20	20	16	12	8	4
HP	–	4 CK	4 CK + 4 D	4 CK + 4 D + 4 R				

*H, harvest time; CK, control group; D, drought treatment; R, rewatering treatment; HP, harvested pots at harvest time.*

### Growth Traits

Height (H, m), basal diameter (BD, cm), and total biomass (TBM, g) were recorded at each harvest time. Four repetitions in each treatment were harvested and separated into roots, stems, and leaves. Then, the samples were oven-dried at 80°C for 48 h and weighed. TBM was calculated as the sum of leaf biomass (LBM, g), stem biomass (SBM, g), and root biomass (RBM, g). The allometric growth was calculated as Šrutek (1997):


lnBD=alnH+b

### Leaf Physiological Traits

Fully expanded and healthy leaves of four repetitions in each treatment of both species were chosen for a net photosynthetic rate (A_P_, μmol m^–2^ s^–1^) measurement on each harvest time, and it was measured *in situ* with an infrared gas analysis system (Li-6800, Li-Cor, Lincoln, NE, United States). The measurements were conducted at 1,000 μmol m^–2^ s^–1^ photosynthetic photo flux density, which was supplied by an external light emitting diode light. Gas-exchange characteristics were measured between 9:00 and 11:00 on sunny days. During the measurement, temperature, relative humidity, and CO_2_ concentration inside the chamber were controlled at 28°C, 50%, and 400 ppm, respectively (Wang et al., 2020; Liu et al., 2021).

### Stem Hydraulic Traits

Xylem midday water potential (Ψ_md_, MPa) of pure planting was measured using one of the seedlings in each plot; Ψ_md_ of mixed planting was estimated by measuring leaf water potential in a pressure chamber (1505D-EXP, PMS Instrument Company, Albany, OR, United States); before measurement, leaves were covered with aluminum foil for 1 h to allow leaf water potential to equilibrate with xylem water potential. Four repetitions of each treatment were measured one by one as soon as they were cut from the seedlings at harvest times after A_P_ measurement.

The stem-specific hydraulic conductivity (K_s_, kg m^–1^ s^–1^ MPa^–1^) was measured at the same time as xylem midday water potential measurement. Four repetitions of each treatment were cut and immersed into degassed water. Subsequently, the samples were transported promptly to the laboratory with the crowns covered with black plastic bags. All leaves and bark were removed, and each segment of *R. pseudoacacia* was 30 cm long, while that of *Q. acutissima* was 20 cm long, to ensure there would never be open vessels (Liu et al., 2020). The segments were connected to a hydraulic conductivity measurement system that contained degassed, filtered 20.0 mmol L^–1^ KCl solution. A 30 cm hydraulic head generated hydrostatic pressure to impel water through the segments. The K_s_ was calculated as follows:


$${\text{K}}_{\text{s}}=\frac{{\text{LQ}}_{\text{m}}}{\text{Sp}},$$

where L is the length of the segment (m), Q_m_ is the mass of the water per unit of time through a segment (kg s^–1^), S is the average cross-sectional area for both ends of the stem (m^2^), and p is the intensity of the water pressure across the segment (MPa) (Liu et al., 2021).

### Statistical Analysis

Data were checked for normality (Shapiro–Wilk test) and homogeneity (Levene test). Four-way analysis of variance (ANOVA) was applied to detect the main effect and interaction of species, planting methods, treatments, and harvest time on plant functional traits. One-way ANOVA was used to test the differences of plant functional traits among different treatments or harvest time within the same species. All ANOVAs were followed by Duncan’s multiple comparison tests at α = 0.05 when significant differences were found. The relationships between height and harvest time, between basal diameter and harvest time, were analyzed by linear regression and quadratic polynomial regression, respectively. The results with significance and larger adjusted coefficient of determination (*R*^2^_adj_) were retained. Data of height and basal diameter were log-transformed, and then, linear regression was performed to find out the allometric growth patterns. All statistical analyses were performed using the SPSS 26 software package (SPSS Inc., Chicago, IL, United States), and all figures were drawn using Origin 2019b (Originlab Co., Northampton, MA, United States).

## Results

### Effects of Species, Planting Methods, Treatments, and Harvest Time

The results of four-way ANOVA show that the main effects of species, planting methods, treatments, and harvest time on plant functional traits are significant (*P* < 0.05). In addition, there were interaction effects of the four factors with plant functional traits (Table 2). In other words, different planting methods, treatments, and harvest time had significant effects on plant hydraulic traits of the two tree species. Then, we compared the differences of plant functional traits of *R. pseudoacacia* and *Q. acutissima* in different planting methods, treatments, and harvest time.

**TABLE 2:** Four-way ANOVA of plant functional traits concerning species (S), planting methods (M), treatments (T), and harvest time (H).

	H	BD	LBM	SBM	RBM	TBM	Ψ_md_	K_s_	A
S	5111.84**	2570.89**	2464.18**	1027.44**	758.32**	2528.17**	106.78**	114.22**	23.72**
M	16.86**	28.58**	321.58**	117.74**	92.13**	306.27**	258.26**	51.77**	402.35**
T	282.77**	243.05**	298.43**	80.85**	61.34**	228.31**	3819.39**	9.07**	736.20**
H	101.43**	43.74**	7.75**	17.36**	17.18**	30.33**	158.73**	2.22	42.47**
S × M	0.84	0.03	187.86**	82.99**	47.71**	187.18**	0.34	65.71**	2.93
S × T	75.82**	105.48**	257.21**	76.05**	45.96**	195.82**	22.36**	3.01	100.07**
S × H	52.07**	21.70**	8.78**	16.90**	12.21**	26.63**	10.54**	0.9	5.45**
M × T	11.16**	26.73**	27.45**	12.98**	6.37**	27.39**	102.06**	3.61*	30.36**
M × H	1.85	0.43	1.48	1.84	2.32*	3.69**	20.75**	2.31*	5.64**
T × H	7.65**	4.20**	7.56**	9.21**	6.66**	16.72**	237.89**	5.54**	46.14**
S × M × T	0.48	42.35**	19.99**	11.35**	3.30*	19.84**	1.25	1.37	4.41*
S × M × H	0.38	0.98	2.13	1.82	2.63*	3.95**	4.26**	1.01	5.69**
S × T × H	3.23**	6.11**	12.50**	14.68**	10.33**	26.80**	9.20**	1.51	6.03**
M × T × H	0.87	2.62*	1.38	1.96	0.7	2.53*	22.49**	1.73	6.56**
S × M × T × H	0.36	2.23*	1.35	2.28*	0.86	2.81*	1.43	0.75	3.99**

H, height, cm; BD, basal diameter, mm; LBM, leaf biomass, g; SBM, stem biomass, g; RBM, root biomass, g; TBM, total biomass, g; Ψ_md_, midday water potential, MPa; K_s_, stem-specific hydraulic conductivity, kg m^–1^ s^–1^ MPa^–1^; A, maximum net photosynthetic rate, μmol m^–2^ s^–1^. Data are F value. *P < 0.05 and **P < 0.01.

### Plant Hydraulic Traits

**FIGURE 1:**
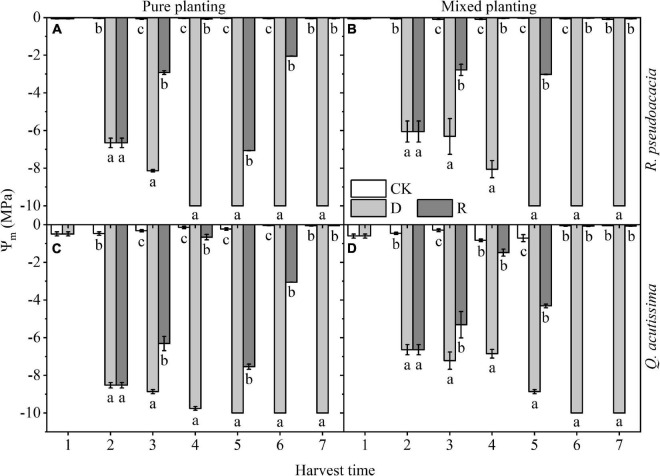
Changes of xylem midday water potential (Ψ_md_, MPa) with harvest time under different planting methods and treatments in *R. pseudoacacia* and *Q. acutissima*. White columns stand for control group (CK), light gray columns stand for drought treatment (D), and gray columns stand for rewatering treatment (R). The first drought-rewatering treatment, 2–4 harvest times; repeated drought-rewatering treatment, 5–7 harvest times. **(A)** *R. pseudoacacia* in pure planting; **(B)** *R. pseudoacacia* in mixed planting; **(C)** *Q. acutissima* in pure planting; and **(D)** *Q. acutissima* in mixed planting. One-way ANOVA followed by Duncan multiple comparison was used to test the differences among treatments; different letters stand for significant difference, *P* < 0.05.

Ψ_md_ was significantly reduced by drought, 10 days after drought, Ψ_md_ reached nearly to –10 MPa, and could recover to the control level (*c.* −0.01 MPa) after rewatering. For *R. pseudoacacia*, Ψ_md_ of RR reached the lowest level (–10 MPa) at Day 30, while that of R_Q_ reached the lowest level at Day 40. For *Q. acutissima*, Ψ_md_ of QQ reached the lowest level at Day 40, while that of Q_R_ reached the lowest level at Day 50. In terms of the two drought-rewatering stages, the water potential decreased by the second drought, which was less than that decreased by the first drought; however, recovery of Ψ_md_ in repeated rewatering treatment of two species was faster than that in the first rewatering treatment, and it was shown that Ψ_md_ at Day 40 was higher than that at Day 10 (*P* < 0.05); it was shorter for repeated rewatering treatment to recover to the control level after rewatering than the first rewatering treatment (Figure 1).

**FIGURE 2:**
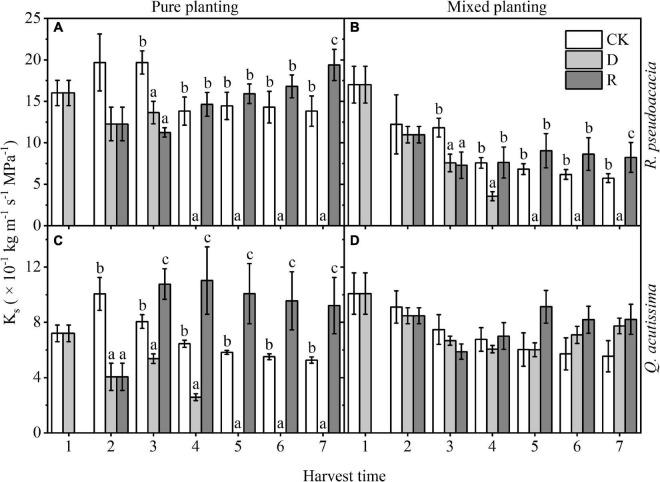
Changes of stem-specific hydraulic conductivity (K_s_, kg m^–1^ s^–1^ MPa^–1^) with harvest time under different planting methods and treatments in *R. pseudoacacia* and *Q. acutissima*. White columns stand for control group (CK), light gray columns stand for drought treatment (D), and gray columns stand for rewatering treatment (R). The first drought-rewater treatment, 2–4 harvest times; repeated drought-rewatering treatment, 5–7 harvest times. **(A)** *R. pseudoacacia* in pure planting; **(B)** *R. pseudoacacia* in mixed planting; **(C)** *Q. acutissima* in pure planting; and **(D)** *Q. acutissima* in mixed planting. One-way ANOVA followed by Duncan multiple comparison was used to test the differences among treatments; different letters stand for significant difference, *P* < 0.05.

K_s_ of RR reached 0, where plant may die, at Day 30; however, K_s_ of R_Q_ reached 0 at Day 40 in *R. pseudoacacia*. Meanwhile, it was higher in rewatering treatment than that in the other two treatments. Compared with pure planting, mixed planting reduced K_s_ (*P* < 0.05). For *Q. acutissima* in QQ, the K_s_ reached 0 at Day 40, while that of Q_R_ never reached 0. In pure planting, K_s_ was significantly higher than that of the other two treatments, but there was no significant difference in mixed planting group (Figure 2).

### Leaf Photosynthetic Traits

**FIGURE 3:**
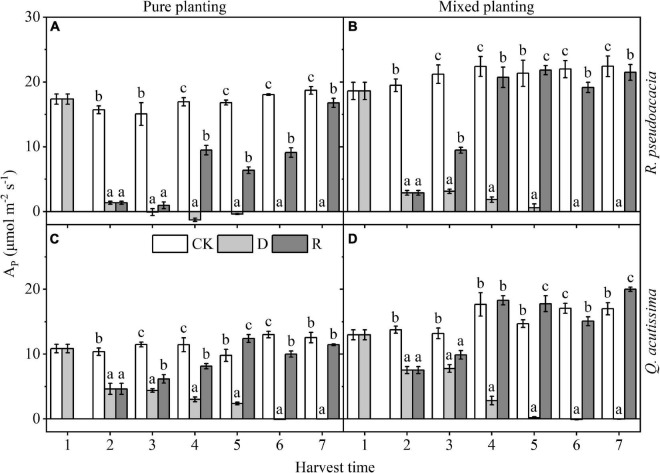
Changes of maximum net photosynthetic rate (A_P_, μmol m^–2^ s^–1^) with harvest time under different planting methods and treatments in *R. pseudoacacia* and *Q. acutissima*. White columns stand for control group (CK), light gray columns stand for drought treatment (D), and gray columns stand for rewatering treatment (R). The first drought-rewater treatment, 2–4 harvest times; repeated drought-rewatering treatment, 5–7 harvest times. **(A)** *R. pseudoacacia* in pure planting; **(B)** *R. pseudoacacia* in mixed planting; **(C)** *Q. acutissima* in pure planting; and **(D)** *Q. acutissima* in mixed planting. One-way ANOVA followed by Duncan multiple comparison was used to test the differences among treatments; different letters stand for significant difference, *P* < 0.05.

For *R. pseudoacacia*, drought reduced A_P_ at first, even less than 0, which recovered rapidly after rewatering, but still significantly less than the control group; A_P_ of R_Q_ was higher than that of RR within the same treatment (*P* < 0.05). For *Q. acutissima*, A_P_ was reduced by drought, but it did not fall below 0. After rewatering, A_P_ recovered rapidly, and A_P_ of QQ could reach the control level, while that of Q_R_ could recover even significantly higher than the control group after rewatering (Figure 3).

### Plant Growth Traits

**FIGURE 4:**
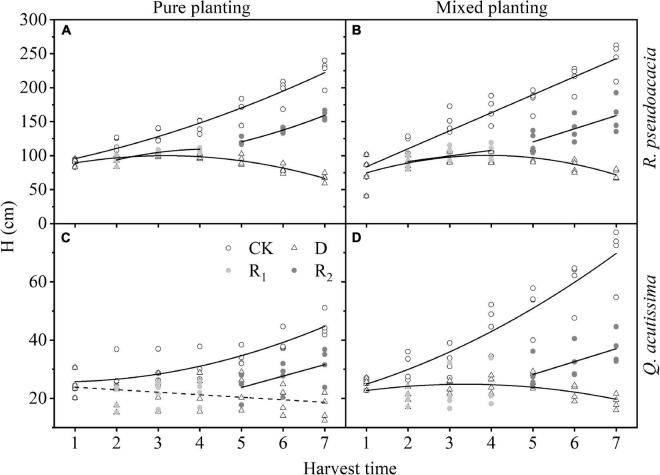
Changes of height (H, cm) with harvest time under different planting methods and treatments in *R*. *pseudoacacia* and *Q. acutissima*. Hollow circles stand for control group (CK), hollow triangles stand for drought treatment (D), light gray circles stand for rewatering treatment (R_1_), and gray circle stands for repeated rewatering treatment (R_2_). The first drought-rewater treatment, 2–4 harvest times; repeated drought-rewatering treatment, 5–7 harvest times. **(A)** *R*. *pseudoacacia* in pure planting; **(B)** *R*. *pseudoacacia* in mixed planting; **(C)** *Q. acutissima* in pure planting; **(D)** and *Q. acutissima* in mixed planting. Lines represent the regression models; solid lines, *P* < 0.05; dash line, *P* < 0.1.

For *R. pseudoacacia*, first, in drought treatment, H increased first and then decreased; the highest H of RR appeared on Day 20, and that of R_Q_ appeared at Day 30. Then, in repeated drought-rewatering treatment, H continued to increase after repeated drought-rewatering, and the growth rate was close to or even higher than the control group especially in repeated rewatering treatment. For *Q. acutissima*, first, the growth rate of H in Q_R_ was higher than that of QQ in control group. In drought treatment, H of QQ began to decrease after drought treatment, while that of Q_R_ first increased after drought treatment, and the highest H appeared at Day 20 and then gradually decreased. Finally, in rewatering treatment, the increase of H in the first rewatering treatment was not obvious, but H in repeated rewatering treatment increased significantly. However, the growth rates were lower than the control group (Figure 4 and Supplementary Table 1).

**FIGURE 5:**
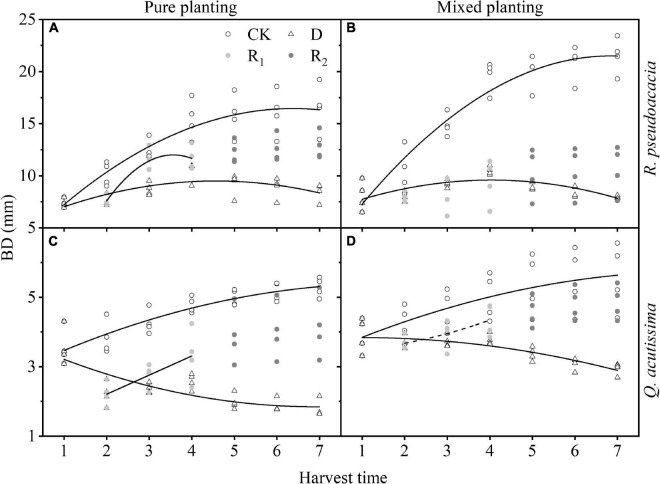
Changes of basal diameter (BD, cm) with harvest time under different planting methods and treatments in *R. pseudoacacia* and *Q. acutissima*. Hollow circles stand for control group (CK), hollow triangles stand for drought treatment (D), light gray circles stand for rewatering treatment (R_1_), and gray circle stands for repeated rewatering treatment (R_2_). The first drought-rewater treatment, 2–4 harvest times; repeated drought-rewatering treatment, 5–7 harvest times. **(A)** *R. pseudoacacia* in pure planting; **(B)** *R. pseudoacacia* in mixed planting; **(C)** *Q. acutissima* in pure planting; **(D)** and *Q. acutissima* in mixed planting. Lines represent the regression models; solid lines, *P* < 0.05; dash line, *P* < 0.1.

For *R. pseudoacacia*, first in CK, BD and its growth rate were significantly increased by mixed planting. Then, in drought treatment, BD increased and then decreased, and the maximum BD of RR appeared at Day 40; however, the maximum BD of R_Q_ appeared at Day 30. Finally, in rewatering treatment, BD of RR increased rapidly during the first rewatering treatment, and the increase rate was even higher than that of the control group. For *Q. acutissima*, first in drought treatment, BD decreased gradually, and the decreasing rate of QQ was fast and then slowed down, while that of Q_R_ was slow and then became faster. Then, in rewatering treatment, BD increased rapidly during the first rewatering treatment, and the increasing rate was close to that of control group, but there was no significant change in BD during repeated rewatering treatment (Figure 5 and Supplementary Table 2).

**FIGURE 6:**
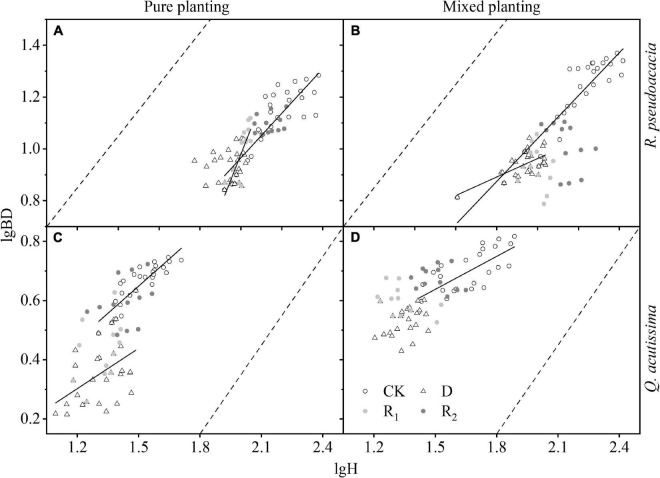
Allometric growth under different planting methods and treatments in *R. pseudoacacia* and *Q. acutissima*. Hollow circles stand for control group (CK), hollow triangles stand for drought treatment (D), light gray circles stand for rewatering treatment (R_1_), and gray circle stands for repeated rewatering treatment (R_2_). **(A)**, *R. pseudoacacia* in pure planting; **(B)** *R. pseudoacacia* in mixed planting; **(C)** *Q. acutissima* in pure planting; **(D)** and *Q. acutissima* in mixed planting. Solid lines represent the regression models, *P* < 0.05; dash lines are lines with slope 1.

For *R. pseudoacacia*, the first rewatering treatment made the allometric growth pattern of RR more inclined to the thickening pattern of basal diameter than control group. However, drought made R_Q_ more inclined to the increase of H; in other treatments, the growth pattern had no obvious trend, and the overall growth rate of H was larger than that of BD. For *Q. acutissima*, Q_R_ was more inclined to the higher H pattern than QQ; in other treatments, the growth pattern had no obvious trend, and the overall growth rate of H was larger than that of BD (Figure 6 and Supplementary Table 3).

**FIGURE 7:**
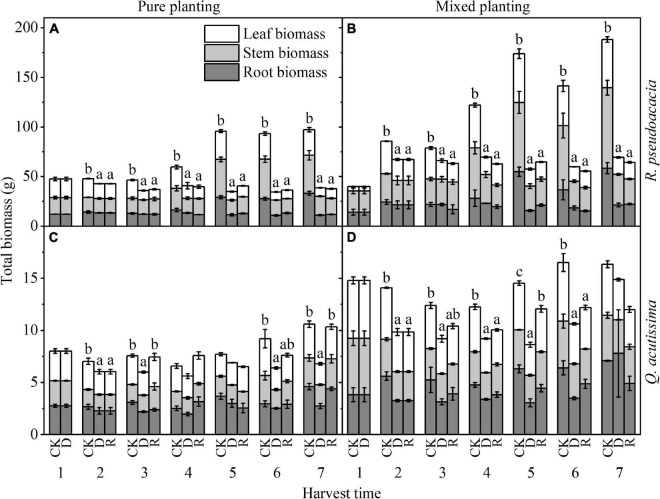
Changes of biomass (g) with harvest time under different planting methods and treatments in *R. pseudoacacia* and *Q. acutissima*. White columns stand for leaf biomass, light gray columns stand for stem biomass, and gray columns stand for root biomass. The first drought-rewater treatment, 2–4 harvest times; repeated drought-rewatering treatment, 5–7 harvest times. **(A)** *R. pseudoacacia* in pure planting; **(B)** *R. pseudoacacia* in mixed planting; **(C)** *Q. acutissima* in pure planting; and **(D)** *Q. acutissima* in mixed planting. One-way ANOVA followed by Duncan multiple comparison was used to test the differences among treatments; different letters stand for significant difference, *P* < 0.05.

For *R. pseudoacacia*, drought made the biomass significantly lower than that of control group. No matter what kind of planting methods and treatments, compared with drought treatment, the biomass of rewatering treatment had no significant change; under the same treatment, the biomass of R_Q_ was higher than that of RR (*P* < 0.05). For *Q. acutissima*, the biomass of drought treatment was little, but it could return to control group after rewatering; under the same treatment, the biomass of Q_R_ was higher than that of QQ (*P* < 0.05) (Figure 7).

## Discussion

### Plant Hydraulic Traits

Different water availability had significant effects on plant hydraulic traits (Figure 1 and Table 2); this may result from the different relative positions of the two tree species on the “isohydric-anisohydric” continuous spectrum, that is, *R. pseudoacacia* is closer to anisohydric plant, and its hydraulic traits respond quickly with the change of water availability, while *Q. acutissima* is closer to the isohydric plant, and its hydraulic traits respond slowly with the change of water availability (Moser et al., 2016; Li et al., 2019, 2020; Liu et al., 2020). The decrease of water potential caused by the second drought was less than that caused by the first drought, and there was no significant difference in Ψ_md_ of the two species between repeated rewatering treatment and control group at the end of repeated rewatering treatment. It may be due to that the first drought made the two species produce stress imprint, so that they could better cope with the repeated drought and recover quickly after rewatering (Munné-Bosch et al., 2003; Jakab et al., 2005; Douma et al., 2017). For the two species, K_s_ of drought treatment decreased gradually, while it maintained at a high level after rewatering. K_s_ of rewatering treatment was almost higher than that of drought treatment, which may be due to the formation of stress imprint after drought stress, so that the two tree species could recover quickly after rewatering and provide enough water for other plant functional traits. Moreover, repeated drought-rewatering did not cause pressure fatigue according to our results. Our results do not find the negative effect of repeated drought-rewatering on plant hydraulic traits of the two tree species. On the contrary, the stress imprint induced by repeated drought-rewatering was conducive to the rapid recovery of the seedlings of the two tree species in the changing environment.

Planting methods had significant effects on plant hydraulic traits (Figures 1, 2 and Table 2). In terms of the relationship between drought and planting methods, the differences in root distribution and structure between *R. pseudoacacia* and *Q. acutissima* may overall reduce the competition for water resource and enhance the drought resistance (Wang and Zhou, 2000). In addition, mixed planting may reduce K_s_ to make the limited water be retained, and it is helpful for the mixed planting *R. pseudoacacia* and *Q. acutissima* to survive under drought stress. However, species in pure planting have similar functional characteristics, resulting in functional redundancy and niche overlap, which may lead to intensified water competition and increased drought stress (Lebourgeois et al., 2013; Pretzsch et al., 2013; Forrester and Pretzsch, 2015; Schwendenmann et al., 2015). The results show that species composition may have a certain effect on drought resistance, and mixed planting could weaken the ecological effect of drought.

### Leaf Photosynthetic Traits

Treatments had significant effects on leaf photosynthetic traits (Table 2 and Figure 3). Combined with the changes of Ψ_md_ (Figure 1) and K_s_ (Figure 2), our results illustrate that both two species formed a stress imprint after the first drought, and when facing the repeated drought, they could better respond to it (Munné-Bosch et al., 2003; Jakab et al., 2005; Douma et al., 2017). In contrast, the two tree species grew rapidly by increasing photosynthesis after rewatering, that is, they adopted the compensatory mechanism. *R. pseudoacacia* adopted an under compensatory mechanism or equal compensatory mechanism (Figures 3A,B), and *Q. acutissima* adopted an equal compensatory mechanism or even over compensatory mechanism (Figures 3C,D; Trlica and Rittenhouse, 1993; Pinkard et al., 2007; Quentin et al., 2012; Wang et al., 2020), to minimize the impact of drought.

Planting methods had significant effects on A_P_ (Table 2). Mixed planting delayed the decrease of A_P_ in drought treatment and increased A_P_ in the control group and rewatering treatment. This is basically consistent with the change of Ψ_md_. Mixed planting prolonged the time for the two species to reach the lowest Ψ_md_ in drought treatment (Figure 1), resulting in the fact that the decrease of A_P_ in drought treatment was delayed. This may come from that repeated drought-rewatering, and different planting methods did not break the water-carbon coupling mechanism of plants (Li et al., 2019, 2020). A_P_ increased as soon as water availability was high. For *R. pseudoacacia*, A_P_ was significantly increased by mixed planting (Figures 3A,B). For *Q. acutissima*, after rewatering, A_P_ of QQ showed an equal compensatory mechanism (Figure 3C), while that of RQ showed an over compensatory mechanism (Figure 3D). Therefore, mixed planting of *R. pseudoacacia* and *Q. acutissima* was not only conducive to drought resistance, but it was also conducive to the recovery and improvement of photosynthetic capacity after rewatering.

### Plant Growth Traits

Treatments had significant effects on H and BD (Table 2). Drought significantly inhibited the growth of H (Figure 4) and BD (Figure 5) of the two tree species, which made the allometric growth pattern different (Figure 6). The allometric growth pattern of *R. pseudoacacia* was not changed by different planting methods, but drought made pure planting and mixed planting present the opposite pattern. In other words, RR presented the BD growth pattern (Figure 6A), while R_Q_ presented the H growth pattern (Figure 6B). It indicated that under drought conditions, RR adopted the survival strategy in the tradeoff between growth-survival strategy. During the fierce intraspecific competition for water resources, RR could increase BD to store more water and nutrients in response to drought stress, while R_Q_ had less competition for water resources (Schwendenmann et al., 2015), and less drought stress was better for *R. pseudoacacia* to get more water by accelerating growth in response to drought. Repeated drought-rewatering increased the growth rate of H of the two tree species (Figure 4) but decreased the growth rate of BD (Figure 5). In terms of plant growth traits, repeated drought did not cause pressure fatigue, while the stress imprint formed by the first drought did not make the two tree species adopt the survival strategy of BD growth mode but take the growth strategy of fast growth.

Planting methods had significant effects on H, BD, and the allometric growth pattern. Our results may be due to the difference in root structure of the two species, resulting in less overall competition for water resource (Schwendenmann et al., 2015). Therefore, under the condition of limited water resources, compared with pure planting, mixed planting reduced the competition for water resources, enhanced drought resistance, and delayed the wilting rate of plant growth (Lebourgeois et al., 2013; Pretzsch et al., 2013). However, pure planting of the two tree species had the same functional characteristics, that is, functional redundancy. The overlap of niches led to the intensification of water competition of *R. pseudoacacia* or *Q. acutissima*, increased the degree of drought, and led to the wilting of plant growth under drought stress. Although the response of mixed species to water use and drought stress is very complex (Forrester and Pretzsch, 2015; De Cáceres et al., 2021), our results show that mixed planting could effectively reduce the competition of water resources and enhance drought resistance in terms of plant growth traits.

Drought had a significant inhibitory effect on BM. After rewatering, BM of *R. pseudoacacia* could not return to the control level (Figures 7A,B), while that of *Q. acutissima* could return to the control level (Figures 7C,D). This may result from that *R. pseudoacacia* is close to the anisohydric plant on “isohydric-anisohydric” continuous spectrum (Moser et al., 2016; Li et al., 2019; Liu et al., 2020). Faced with drought, the biomass accumulation stopped, due to the rapid decline of plant hydraulic traits (Figures 1A,B) and the rapid decline of A_P_ (Figures 3A,B), while BM increased slowly after rewatering due to the compensatory mechanism of photosynthesis, especially in RR. However, *Q. acutissima* was close to the anisohydric plant on “isohydric-anisohydric” continuous spectrum (Moser et al., 2016; Li et al., 2019; Liu et al., 2020). Faced with drought, plant hydraulic traits decreased slowly (Figures 1C,D), leading to a slow decrease in photosynthesis (Figures 3C,D). After rewatering, BM increased rapidly due to the over compensatory mechanism of photosynthesis, especially in Q_R_. Planting methods have significant effects on BM (Table 2). Mixed planting increased the BM of the two species in each treatment (Figures 7B,D), which may result from the more reasonable allocation of resources, reducing the competition between the two species and conserving water and soil (Schwendenmann et al., 2015).

### Response of *R. pseudoacacia* and *Q. acutissima*

Drought had a significant effect on the plant functional traits of the two tree species under different planting methods. It decreased Ψ_md_, K_s_, and A_P_ of the two tree species, and ultimately reduced H, BD, and BM. Mixed planting could reduce the competition of light, water, space, and other resources, making the limited resources more reasonable allocation, so as to weaken the inhibition of drought on the two tree species to a certain extent. After rewatering, plant functional traits were recovered. Repeated drought-rewatering did not make the two tree species produce pressure fatigue. On the contrary, it made the two tree species form stress imprint, that is, the storage of stress-resistant substances through physiological and biochemical responses. The stress imprint could better respond to drought when faced with drought stress again. After rewatering, Ψ_md_ quickly recovered, and photosynthesis of *R. pseudoacacia* adopted compensatory strategies. Therefore, the BM was accumulated. Photosynthesis of *Q. acutissima* was overcompensated to make the BM close to or even reach the level without drought. Therefore, the combination of the two species and repeated drought-rewatering can improve the overall drought resistance ability to a certain extent.

Through the experiment of repeated drought-rewatering of *R. pseudoacacia* and *Q. acutissima* under pure and mixed planting, it is found that mixed planting of the two tree species could weaken the inhibition of drought to a certain extent; repeated drought-rewatering did not cause pressure fatigue to the two species but made them form stress imprint, which can quickly respond to repeated drought and recover quickly after rewatering. Our research suggested that the mixed planting of the two tree species could better adapt to repeated drought-rewatering under global climate change.

## Data Availability Statement

The original contributions presented in the study are included in the article/Supplementary Material, further inquiries can be directed to the corresponding author/s.

## Author Contributions

XL, HW, PZ, PF, ND, and RW proposed the study. XL conducted the field and laboratory measurements and analyzed the data. HW, PZ, and RW designed the experiment and secured the funding. QZ, MS, and NW helped in the laboratory measurements and data analysis. PW and KC helped in the data analysis. XL wrote the manuscript that was intensively edited by all authors. All authors contributed to the article and approved the submitted version.

## Conflict of Interest

The authors declare that the research was conducted in the absence of any commercial or financial relationships that could be construed as a potential conflict of interest.

## Publisher’s Note

All claims expressed in this article are solely those of the authors and do not necessarily represent those of their affiliated organizations, or those of the publisher, the editors and the reviewers. Any product that may be evaluated in this article, or claim that may be made by its manufacturer, is not guaranteed or endorsed by the publisher.
