# Supplementary material for: Physiological Responses of Robinia pseudoacacia and Quercus acutissima Seedlings to Repeated Drought-Rewatering Under Different Planting Methods
Source: Front Plant Sci. 2021 Dec 6;12:760510. doi: 10.3389/fpls.2021.760510 (PMC8685255; doi:10.3389/fpls.2021.760510)
Supplement: Supplementary file 1 [file Data_Sheet_1.docx]

**Supplementary Table 1** Regression analysis of height (H, cm) and harvest time under different planting methods and treatments in *R*. *pseudoacacia* and *Q. acutissima*.

| Species | Planting methods | Treatments | ax^2^ | bx | c | *R*^2^ | SA | H_max_ | H_min_ |
| --- | --- | --- | --- | --- | --- | --- | --- | --- | --- |
| *R. pseudoacacia* | Pure planting | CK | 1.22 | 11.37 | 83.01 | **0.97**** | –4.66 | 7 | 1 |
|  |  | D | –2.28 | 14.50 | 76.92 | **0.80**** | 3.18 | 3 | 7 |
|  |  | R_1_ | –3.14 | 26.81 | 52.56 | **0.69**** | 4.27 | 4 | 2 |
|  |  | R_2_ | 2.16 | –6.53 | 98.75 | **0.92**** | 1.51 | 7 | 5 |
|  | Mixed planting | CK | - | 21.51 | 77.07 | **0.89**** | - | 7 | 1 |
|  |  | D | –3.02 | 23.71 | 53.88 | **0.58**** | 3.93 | 4 | 7 |
|  |  | R_1_ | - | 8.16 | 75.10 | **0.34*** | - | 4 | 2 |
|  |  | R_2_ | - | 19.38 | 23.27 | **0.45*** | - | 7 | 5 |
| *Q. acutissima* | Pure planting | CK | 0.47 | –0.56 | 25.85 | **0.71**** | 0.60 | 7 | 1 |
|  |  | D | - | –0.87 | 24.71 | 0.12 | - | 1 | 7 |
|  |  | R_1_ | - | - | - | - | - | - | - |
|  |  | R_2_ | - | 0.47 | 2.10 | **0.35*** | - | 7 | 5 |
|  | Mixed planting | CK | 0.49 | 3.59 | 20.81 | **0.85**** | –3.66 | 7 | 1 |
|  |  | D | –0.39 | 2.63 | 20.44 | **0.24*** | 3.37 | 3 | 7 |
|  |  | R_1_ | - | - | - | - | - | - | - |
|  |  | R_2_ | - | 4.43 | 6.08 | **0.36*** | - | 7 | 5 |

Note: CK, control group; D, drought treatment; R_1_, rewater group; R_2_, repeated rewater group; ax^2^, second-order coefficient; bx, first-order coefficient; c, constant; SA, symmetry axis; H_max_, harvest time corresponding to maximum plant height; H_min_, harvest time corresponding to minimum plant height; the black bold font numbers in Row R^2^ indicate significance, *, *P* < 0.05, * *, *P* < 0.01; the regular font number indicates *P* < 0.1; "-" indicates no such item.

**Supplementary Table 2** Regression analysis of basal diameter (BD, mm) and harvest time under different planting methods and treatments in *R*. *pseudoacacia* and *Q. acutissima*.

| Species | Planting methods | Treatments | ax^2^ | bx | c | *R*^2^ | SA | BD_max_ | BD_min_ |
| --- | --- | --- | --- | --- | --- | --- | --- | --- | --- |
| *R. pseudoacacia* | Pure planting | CK | –0.32 | 4.07 | 3.48 | **0.82**** | 6.36 | 6 | 1 |
|  |  | D | –0.19 | 1.78 | 5.44 | **0.46**** | 4.68 | 5 | 1 |
|  |  | R_1_ | –1.81 | 12.87 | –10.95 | **0.84**** | 3.56 | 4 | 2 |
|  |  | R_2_ | - | - | - | - | - |  |  |
|  | Mixed planting | CK | –0.42 | 5.71 | 1.97 | **0.90**** | 6.80 | 7 | 1 |
|  |  | D | –0.20 | 1.62 | 6.32 | **0.47**** | 4.05 | 4 | 1 |
|  |  | R_1_ | - | - | - | - | - | - | - |
|  |  | R_2_ | - | - | - | - | - | - | - |
| *Q. acutissima* | Pureplanting | CK | –0.03 | 0.57 | 2.93 | **0.79**** | 9.50 | 7 | 1 |
|  |  | D | 0.04 | -0.54 | 3.71 | **0.60**** | 6.75 | 1 | 7 |
|  |  | R_1_ | - | 0.55 | 1.11 | **0.50*** | - | 4 | 2 |
|  |  | R_2_ | - | - | - | - | - | - | - |
|  | Mixed planting | CK | –0.03 | 0.53 | 3.34 | **0.47**** | 8.83 | 7 | 1 |
|  |  | D | –0.03 | 0.05 | 3.82 | **0.65**** | 0.83 | 1 | 7 |
|  |  | R_1_ | 0.05 | 0.00 | 3.46 | 0.41 | 0.00 | 4 | 2 |
|  |  | R_2_ | - | - | - | - | - | - | - |

Note: CK, control group; D, drought treatment; R_1_, rewater group; R_2_, repeated rewater group; ax^2^, second-order coefficient; bx, first-order coefficient; c, constant; SA, symmetry axis; BD_max_, harvest time corresponding to maximum basal diameter; BD_min_, harvest time corresponding to minimum basal diameter; the black bold font numbers in Row R^2^ indicate significance, *, *P* < 0.05, * *, *P* < 0.01; the regular font number indicates *P* < 0.1; "-" indicates no such item.

**Supplementary Table 3** Linear regression analysis of allometric growth under different planting methods and treatments in *R*. *pseudoacacia* and *Q. acutissima*.

| Species | Planting methods | Treatments | Slope | Intercept | R^2^ |
| --- | --- | --- | --- | --- | --- |
| *R. pseudoacacia* | Pure planting | CK | 0.86 | –0.76 | **0.73**** |
|  |  | D | - | - | - |
|  |  | R_1_ | 2.01 | -3.05 | **0.6**** |
|  |  | R_2_ | - | - | - |
|  | Mixed planting | CK | 0.83 | –0.62 | **0.85**** |
|  |  | D | 0.36 | 0.25 | **0.4**** |
|  |  | R_1_ | - | - | - |
|  |  | R_2_ | - | - | - |
| *Q. acutissima* | Pure planting | CK | 0.61 | –0.27 | **0.77**** |
|  |  | D | 0.45 | –0.24 | **0.22*** |
|  |  | R_1_ | - | - | - |
|  |  | R_2_ | - | - | - |
|  | Mixed planting | CK | 0.37 | 0.08 | **0.54**** |
|  |  | D | - | - | - |
|  |  | R_1_ | - | - | - |
|  |  | R_2_ | - | - | - |

Note: CK, control group; D, drought treatment; R_1_, rewater group; R_2_, repeated rewater group; the black bold font numbers in Row R^2^ indicate significance, *, *P* < 0.05, * *, *P* < 0.01; the regular font number indicates *P* < 0.1; "-" indicates no such item.
